# The Medical World

**Published:** 1871-08

**Authors:** 


*The Medical World.*

This is the title of a new monthly medical journal which has made its appearance in New York, edited by Reuben A. Vance, M. D., and published by

Wm. Baldwin & Co., 21 Park Row. The first number was issued on the first of July, and contained many well written and well selected articles.

